# The Impact of Health Information Exchange on In-Hospital and Postdischarge Mortality in Older Adults with Alzheimer Disease Readmitted to a Different Hospital Within 30 Days of Discharge: Cohort Study of Medicare Beneficiaries

**DOI:** 10.2196/41936

**Published:** 2023-03-10

**Authors:** Sara Turbow, Camille P Vaughan, Steven D Culler, Kenneth W Hepburn, Kimberly J Rask, Molly M Perkins, Carolyn K Clevenger, Mohammed K Ali

**Affiliations:** 1 Division of General Internal Medicine Department of Medicine Emory University School of Medicine Atlanta, GA United States; 2 Department of Family & Preventive Medicine Emory University School of Medicine Atlanta, GA United States; 3 Division of Geriatrics & Gerontology Department of Medicine Emory University School of Medicine Atlanta, GA United States; 4 Birmingham/Atlanta Geriatric Research Education and Clinical Center Department of Veterans Affairs Atlanta, GA United States; 5 Department of Health Policy and Management Rollins School of Public Health Emory University Atlanta, GA United States; 6 Nell Hodgson Woodruff School of Nursing Emory University Atlanta, GA United States; 7 Alliant Health Atlanta, GA United States; 8 Hubert Department of Global Health Rollins School of Public Health Emory University Atlanta, GA United States

**Keywords:** readmissions, care fragmentation, health information exchange, mortality, Alzheimer disease, electronic health information, information sharing, older adults, information exchange, hospital system, health informatics

## Abstract

**Background:**

Although electronic health information sharing is expanding nationally, it is unclear whether electronic health information sharing improves patient outcomes, particularly for patients who are at the highest risk of communication challenges, such as older adults with Alzheimer disease.

**Objective:**

To determine the association between hospital-level health information exchange (HIE) participation and in-hospital or postdischarge mortality among Medicare beneficiaries with Alzheimer disease or 30-day readmissions to a different hospital following an admission for one of several common conditions.

**Methods:**

This was a cohort study of Medicare beneficiaries with Alzheimer disease who had one or more 30-day readmissions in 2018 following an initial admission for select Hospital Readmission Reduction Program conditions (acute myocardial infarction, congestive heart failure, chronic obstructive pulmonary disease, and pneumonia) or common reasons for hospitalization among older adults with Alzheimer disease (dehydration, syncope, urinary tract infection, or behavioral issues). Using unadjusted and adjusted logistic regression, we examined the association between electronic information sharing and in-hospital mortality during the readmission or mortality in the 30 days following the readmission.

**Results:**

A total of 28,946 admission-readmission pairs were included. Beneficiaries with same-hospital readmissions were older (aged 81.1, SD 8.6 years) than beneficiaries with readmissions to different hospitals (age range 79.8-80.3 years, *P*<.001). Compared to admissions and readmissions to the same hospital, beneficiaries who had a readmission to a different hospital that shared an HIE with the admission hospital had 39% lower odds of dying during the readmission (adjusted odds ratio [AOR] 0.61, 95% CI 0.39-0.95). There were no differences in in-hospital mortality observed for admission-readmission pairs to different hospitals that participated in different HIEs (AOR 1.02, 95% CI 0.82-1.28) or to different hospitals where one or both hospitals did not participate in HIE (AOR 1.25, 95% CI 0.93-1.68), and there was no association between information sharing and postdischarge mortality.

**Conclusions:**

These results indicate that information sharing between unrelated hospitals via a shared HIE may be associated with lower in-hospital, but not postdischarge, mortality for older adults with Alzheimer disease. In-hospital mortality during a readmission to a different hospital was higher if the admission and readmission hospitals participated in different HIEs or if one or both hospitals did not participate in an HIE. Limitations of this analysis include that HIE participation was measured at the hospital level, rather than at the provider level. This study provides some evidence that HIEs can improve care for vulnerable populations receiving acute care from different hospitals.

## Introduction

Hospitalizations frequently increase during the final months of an older person’s life: two-thirds of Medicare fee-for-service beneficiaries are hospitalized in the final 6 months of life and 25% have multiple hospitalizations [1,2]. The transitions of care that occur at the end of life can lead to more readmissions [3], disruptions that may be further exacerbated by the presence of dementia. Previous work has shown that cognitive impairment is associated with decreases in both the quality of care a person receives in the hospital [4,5] and the patient’s and their caregiver’s ability to follow discharge instructions [6].

One underexamined factor that may worsen outcomes following hospitalizations in older adults is interhospital fragmentation of care, which occurs when an individual is readmitted to a hospital different than the one from which they were initially discharged. This happens in approximately 25% of all readmissions nationally and is associated with poor patient outcomes, including higher in-hospital mortality and longer lengths of stay [7-10]. Information discontinuity is one potential driver of poor outcomes in fragmented readmissions: because a patient’s medical record may not be available at the readmission hospital, the care team may be making decisions with incomplete clinical information. Health information exchanges (HIEs), data systems in which health information is electronically shared between settings of care [11,12], are a potential solution to information discontinuity and associated challenges present in fragmented readmissions.

Previous work in general adult patient populations suggests that HIE availability in the inpatient setting may be associated with fewer readmissions [13,14], particularly fragmented readmissions [15], and may be associated with a reduction in repeat laboratory and imaging tests [16-20]. If the improvement in these metrics is due to improved care coordination attributable to information obtained from the HIE, we hypothesize that these positive impacts would extend to outcomes during and following hospitalizations as well. Those with cognitive impairment may be especially vulnerable in fragmented readmissions where outside clinical information is not available. The goal of this study was to measure the association between electronic information sharing, in-hospital mortality, and mortality in the 30 days following hospital readmission among Medicare beneficiaries with Alzheimer disease (AD) initially admitted for common conditions and then readmitted to a different hospital. This information will contribute to our understanding of the impact and limitations of HIEs as tools to mitigate information discontinuity across providers. HIEs have the potential to improve care for vulnerable populations, such as older adults with AD, but this potential is limited if the 2 hospitals do not share an HIE or if one does not participate.

## Methods

### Study Design

**Figure 1:**
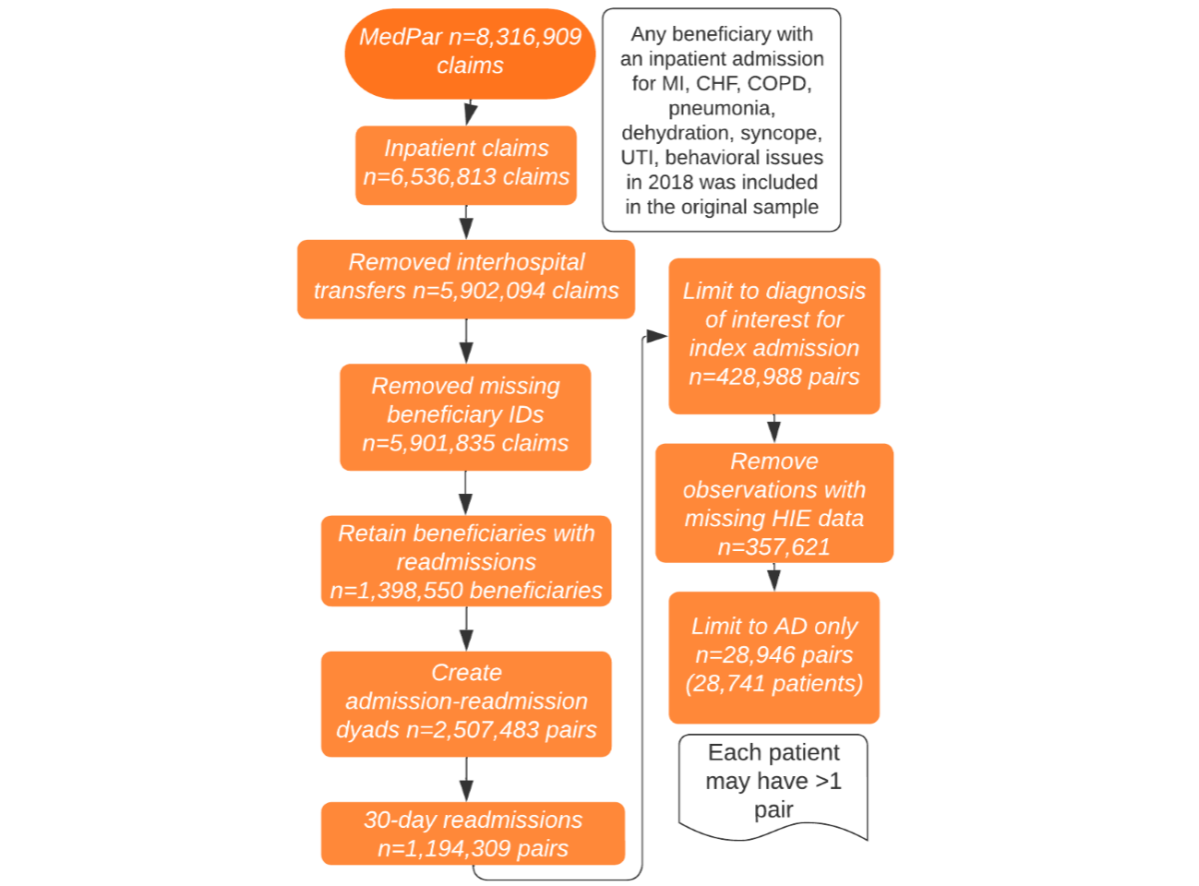
Sample development. AD: Alzheimer disease; CHF: congestive heart failure; COPD: chronic obstructive pulmonary disease; HIE: health information exchange; MI: myocardial infarction; UTI: urinary tract infection.

We analyzed data from a longitudinal cohort of all Medicare beneficiaries in 2018 with a hospital admission for acute myocardial infarction (AMI), congestive heart failure (CHF), chronic obstructive pulmonary disease (COPD), pneumonia, dehydration, syncope, urinary tract infection (UTI), or behavioral issues and a subsequent readmission within 30 days for any reason (Figure 1). The objective of this study is to measure the association between electronic information sharing, in-hospital mortality, and mortality in the 30 days following hospital readmission to a different hospital than the beneficiary was previously discharged from.

### Data Sources

The primary data source for this analysis was the 2018 Medicare Provider Analysis and Review (MedPar) file, which includes inpatient Medicare claims. Additional clinical characteristics were obtained from the 2018 Medicare Master Beneficiary Summary and the Chronic Conditions Segment files. Hospital characteristics were obtained from the 2018 American Hospital Association (AHA) Annual Survey [21]. HIE participation was obtained from the AHA Information Technology (IT) Supplement from 2017 and 2018 [22,23].

### Patients

Inpatient claims from Medicare beneficiaries who had a hospital admission for AMI, CHF, COPD, pneumonia, dehydration, syncope, UTI, or behavioral issues in 2018 were obtained from the Centers for Medicare and Medicaid Services (CMS). Multimedia Appendix 1 shows the International Classification of Diseases–10 codes and diagnosis related group codes used. These conditions were chosen because they are either conditions in the Hospital Readmissions Reduction Program (HRRP) [24]—conditions, including AMI, CHF, COPD, and pneumonia, identified by the CMS as having a high risk of readmission [25]—or are common causes of hospitalization among older adults, particularly those with AD [26,27]. While the index admission was for one of the above reasons, the readmission could be for any reason. We excluded beneficiaries who did not have a readmission in the data set, claims with missing beneficiary identification numbers, and claims that represented admissions resulting from an interhospital transfer. The unit of observation was transformed from a claim to an admission-readmission pair. If a beneficiary had more than 2 hospital admissions, multiple admission-readmission pairs were created (Multimedia Appendix 2 provides examples). We then removed all admission-readmission pairs in which the time from discharge to readmission was over 30 days. The analysis was limited to beneficiaries who were listed as having a diagnosis of AD in the chronic conditions segment; this data source includes diagnoses from 1999 onward [28].

### Primary Exposure: Type of Information Sharing

We categorized electronic information sharing based on the availability of electronic information exchange between a beneficiary’s admission and readmission hospitals based on the AHA Annual Survey and IT Supplement. The IT Supplement asks, “Please indicate your level of participation in a state, regional and/or local health information exchange or health information organization.” Answers could be “do not know,” “not operational,” “operational...we are not participating,” or “operational...we are participating and actively exchanging data.” The IT survey additionally asks, “Which of the following national health information exchange networks does your hospital participate in?” Several options are provided, including “your [electronic health record] vendor’s network which enables exchange with vendor’s other users” and “other.” If a hospital answered “other” and provided a free-text answer to describe the HIE they participated in, their answers were recoded to be comparable between admission and readmission hospitals. If a hospital had different or missing answers across the 2 years of data, we imputed the answer reflecting their highest level of participation. Hospitals that did not respond to the HIE survey in 2017 and 2018 or who did not respond to the participation in HIE questions were excluded from the analysis.

We classified information sharing into 4 distinct categories: same-hospital readmissions and 3 categories of information exchange between different hospitals with fragmented readmissions. The first type of information sharing was “same-hospital readmission.” In this scenario, all the information from the initial admission should be readily available in the patient’s medical record during their readmission, so there is no expectation that the hospital’s HIE status would have an impact on the quality of care they received. Because these patients did not experience care fragmentation, this group served as the reference group for subsequent analyses. Second, patients could have a fragmented readmission to different hospitals in which both hospitals participate in the same HIE based on their answers to the AHA IT survey (ie, “fragmented/same HIE”). In this scenario, the information from the index admission is available to the readmission hospital via the HIE. The third type of information sharing was a fragmented readmission to different hospitals in which each hospital participated in an HIE, but the HIEs were different between the admission and readmission hospitals (ie, “fragmented/different HIE”). This category captures hospitals that participate in an HIE because they may be different than hospitals that do not participate in HIE, but in this scenario, there is no clear method of electronic information exchange between the admission and readmission hospitals, so information from the index admission is not available to clinicians at the readmission hospital. The final category was “no information shared”: fragmented readmissions to different hospitals in which one or both hospitals indicated on the survey that they did not participate in an HIE (ie, “fragmented/no HIE”). Because a beneficiary could have multiple admission-readmission pairs in this analysis, they could have a pair in more than one category of information sharing. In both the fragmented/different HIE and the fragmented/no HIE categories, there is less expectation that participation in different HIEs by both hospitals or participation in an HIE by only one hospital would have an impact on the quality of care.

### Outcomes: In-Hospital and 30-Day Postdischarge Mortality

The outcomes of interest for this study were all-cause in-hospital mortality and all-cause mortality in the 30 days following discharge from the readmission (among beneficiaries who survived their readmission). We used the death date of each beneficiary, where applicable, to determine vital status and when the beneficiary died.

### Covariates

We included several beneficiary demographic and clinical characteristics, as well as hospital characteristics, in our models. Demographic characteristics included the beneficiary’s age, sex, and race (White, Black, or other). Clinical characteristics included a frailty score, the number of chronic conditions, the reason for readmission to the hospital, and if the beneficiary was admitted to the intensive care unit (ICU) during the readmission. The frailty score ranges from 0 to 1, with a higher score indicating greater frailty; it was calculated using a deficit-accumulation model using 93 claims-based variables [29,30]. The number of chronic conditions was measured by counting the number of chronic conditions with which the beneficiary was ever diagnosed in the Chronic Conditions Supplement, which reports 27 chronic conditions [28]. Reason for readmission was divided into 9 categories, based on the 8 categories of interest identified for index admissions and 1 “other reason” category.

Hospital characteristics included hospital size (<500 or ≥500 beds), ownership (government, religious, nonprofit, or for-profit), hospital type (general medical/surgical, or other), urban/rural status of the hospital (metropolitan, micropolitan, or rural), and if the hospital was a teaching hospital or not. Urban/rural status was identified via the Rural Urban Commuting Area codes of the hospital [31]. Hospitals were classified as teaching hospitals if they reported that they had programs accredited by the American Council of Graduate Medical Education, the American Osteopathic Association, or the Council of Teaching Hospitals, or if they were affiliated with a medical school. Hospitals were categorized as either general medical/surgical or “other,” which included specialty hospitals [21].

### Analytic Approach

Univariate statistics were used to describe and compare clinical and demographic characteristics between admission-readmission pairs across categories of information sharing. Hospital characteristics by HIE status across all hospitals that responded to the AHA Annual Survey and AHA IT survey were also assessed.

To evaluate whether electronic information sharing via HIE between admission and readmission hospitals was associated with in-hospital or postdischarge mortality, we performed unadjusted and adjusted logistic regressions. Regression analyses were adjusted separately for patient demographics and clinical characteristics (age, sex, race, frailty score, number of chronic conditions, reason for readmission, and ICU use during readmission) and for readmission hospital characteristics (number of beds, ownership, hospital type, urban/rural location, and teaching status). Regressions included hospital fixed effects to adjust for unmeasured differences between hospitals. Robust standard errors clustered at the hospital level were used.

We also completed several sensitivity analyses. First, to test the influence of rural hospitals, which may have different market structures than micropolitan or urban hospitals, we removed readmissions to rural hospitals. Second, we limited the analysis to beneficiaries who did not have an ICU stay during their index hospital admission to select for patients at lower risk for death. Third, to determine if HIE use might have a stronger association with mortality in patients at higher risk for mortality, we calculated the probability of dying within 90 days following hospital discharge and analyzed only beneficiaries with a 90-day mortality probability of >0.25. We also created propensity-score matched cohorts on the odds of 30-day postdischarge mortality using optimal matching without replacement; this was done to balance the odds of dying across information-sharing categories. Finally, to test if patients who are frequently admitted to the same hospital had a disproportionate influence on the results of same hospital admission-readmission pairs, we limited the same-hospital category to only the first pair for each beneficiary. Analyses were completed in SAS (version 9.4; SAS Institute) and Stata (version 17; Stata Corp).

### Ethical Considerations

This study was approved by the Institutional Review Board of Emory University School of Medicine (#00000108) and funded by the National Institute on Aging of the National Institutes of Health (K23AG065505).

## Results

### Participant Characteristics

The initial sample had 8,316,909 claims. We removed noninpatient claims, interhospital transfers, and observations missing beneficiary identification numbers. We then created admission-readmission pairs; after limiting the pairs to readmissions within 30 days, limiting the pairs to index admissions for the initial diagnoses of interest, and removing pairs in which the beneficiary was listed as deceased after their index admission, we had 428,988 pairs (including 279,729 unique patients). Next, we removed observations with missing HIE data due to nonresponse to the AHA IT survey or nonresponse to the HIE questions on the AHA IT survey (n=71,367, 16.6% of pairs were removed). Of the remaining 357,621 pairs, 8.1% were for beneficiaries with AD (n=28,946 pairs comprising 28,741 unique patients), representing the final sample. Full details of the sample development can be found in Figure 1.

Beneficiaries with same-hospital readmissions were older (aged 81.1, SD 8.6 years) than beneficiaries with readmissions to a different hospital (age range 79.8-80.3 years, *P*<.001; Table 1). There were no differences in frailty score or chronic condition count across the categories of information sharing. While 49.9% of hospitals that responded to the AHA and AHA IT surveys reported participating in an HIE (Multimedia Appendix 3), only 2.1% (601/28,946) of admission-readmission pairs were to hospitals that shared an HIE. Overall, 5.9% (1704/28,946) of beneficiaries died during their readmission, and 19.6% (5667/28,946) died in the 30 days following hospital readmission (Table 1).

**Table 1:** Demographic and clinical information of admission-readmission pairs among Medicare beneficiaries with Alzheimer disease in 2018.

			Total (n=28,946)		Same-hospital readmission (n=24,952)		Fragmented readmission						*P* value	
							Same HIE^a^ (n=601)	Different HIEs (n=2105)		No HIE (n=1288)				
Age (years), mean (SD)			80.9 (8.6)		81.1 (8.6)		80.3 (8.7)	80.1 (8.6)		79.8 (8.4)		<.001		
**Sex, n (%)**														.10
	Female	16,163 (55.8)		14,004 (56.1)		329 (54.7)		1142 (54.2)	688 (53.4)					
	Male	12,783 (44.2)		10,948 (43.9)		272 (45.3)		963 (45.7)	600 (46.6)					
**Race, n (%)**														<.001
	White	24,097 (83.2)		20,976 (84.1)		467 (77.7)		1644 (78.1)	1010 (78.4)					
	Black	3341 (11.5)		2692 (10.8)		111 (18.5)		335 (15.9)	203 (15.8)					
	Other	1508 (5.2)		1284 (5.1)		23 (3.8)		126 (6)	75 (5.8)					
**Urban/rural status, n (%)**														<.001
	Metropolitan	25,110 (87.1)		21,641 (87)		526 (87.8)		1878 (89.5)	1065 (82.9)					
	Micropolitan	2748 (9.5)		2424 (9.7)		43 (7.2)		131 (6.2)	150 (11.7)					
	Rural	985 (3.4)		797 (3.2)		30 (5)		89 (4.2)	69 (5.4)					
Frailty score, mean (SD)			0.20 (0.05)		0.20 (0.05)		0.20 (0.05)	0.21 (0.05)		0.20 (0.05)		.45		
Chronic condition count, mean (SD)			20.7 (6.2)		20.7 (6.2)		20.4 (6.0)	20.5 (6.3)		20.7 (6.2)		.24		
**Reason for admission, n (%)**														<.001
	MI^b^	2451 (8.5)		2047 (8.2)		57 (9.5)		209 (9.9)	138 (10.7)					
	CHF^c^	12,103 (41.8)		10,496 (42.1)		266 (44.3)		834 (39.6)	507 (39.4)					
	COPD^d^	3916 (13.5)		3405 (13.6)		76 (12.6)		266 (12.6)	169 (13.1)					
	Pneumonia	4716 (16.3)		4097 (16.4)		91 (15.1)		336 (16)	192 (14.9)					
	Dehydration	1311 (4.5)		1101 (4.4)		33 (5.5)		112 (5.3)	65 (5)					
	Syncope	459 (1.6)		364 (1.5)		13 (2.2)		54 (2.6)	28 (2.2)					
	UTI^e^	3954 (13.7)		3411 (13.7)		65 (10.8)		290 (13.8)	188 (14.6)					
	Behavioral issues	36 (0.1)		31 (0.1)		0 (0)		4 (0.2)	1 (0.01)					
**Reason for readmission, n (%)**														<.001
	MI	651 (2.2)		516 (2.1)		29 (4.8)		63 (3)	43 (3.3)					
	CHF	6362 (22)		5584 (22.4)		125 (20.8)		405 (19.2)	248 (19.2)					
	COPD	1743 (6)		1551 (6.2)		27 (4.5)		97 (4.6)	68 (5.3)					
	Pneumonia	1376 (4.7)		1238 (5)		18 (3)		74 (3.5)	46 (3.6)					
	Dehydration	434 (1.5)		379 (1.5)		6 (1)		34 (1.6)	15 (1.2)					
	Syncope	132 (0.5)		111 (0.4)		4 (0.7)		9 (0.4)	8 (0.6)					
	UTI	936 (3.2)		838 (3.4)		8 (1.3)		55 (2.6)	35 (2.7)					
	Behavioral issues	7 (0.02)		6 (0.02)		0 (0)		0 (0)	1 (0.01)					
	Other	17305 (59.8)		14,729 (59)		384 (63.9)		1368 (65)	824 (64)					
ICU^f^ stay admission			8651 (29.9)		7483 (30)		153 (25.5)	603 (28.6)		412 (32)		.02		
ICU stay readmission			9727 (33.6)		8269 (33.1)		202 (33.6)	772 (36.7)		484 (37.6)		<.001		
**Readmission hospital number of beds, n (%)**														<.001
	<500 beds	21,789 (75.5)		18,909 (76.1)		401 (66.8)		1505 (71.7)	974 (76.1)					
	≥500 beds	7051 (24.5)		5953 (23.9)		199 (33.2)		593 (28.3)	306 (23.9)					
**Readmission hospital ownership, n (%)**														<.001
	Government	2911 (10.1)		2483 (10)		63 (10.5)		204 (9.7)	161 (12.6)					
	Religious	3444 (11.9)		2997 (12.1)		67 (11.2)		260 (12.4)	120 (9.4)					
	Nonprofit	18,746 (65)		16,288 (65.5)		439 (73.2)		1271 (60.6)	748 (58.5)					
	For-profit	3724 (12.9)		3080 (12.4)		31 (5.2)		363 (17.3)	250 (19.5)					
**Readmission hospital type, n (%)**														<.001
	General medical/surgical	28,612 (99.3)		24,799 (99.8)		582 (97.2)		2026 (96.8)	1205 (94.4)					
	Other	194 (0.7)		38 (0.1)		17 (2.8)		67 (3.2)	72 (5.6)					
Readmission hospital was a teaching hospital			20,937 (72.6)		17,959 (72.2)		469 (78.2)	1640 (78.2)		869 (67.9)		<.001		
Died during readmission			1704 (5.9)		1459 (5.8)		29 (4.8)	130 (6.2)		86 (6.7)		.38		
30-Day postdischarge mortality^g^			5667 (19.6)		4856 (19.5)		118 (19.6)	428 (20.3)		265 (20.6)		.62		

^a^HIE: health information exchange.

^b^MI: myocardial infarction.

^c^CHF: congestive heart failure.

^d^COPD: chronic obstructive pulmonary disease.

^e^UTI: urinary tract infection.

^f^ICU: intensive care unit.

^g^Among beneficiaries who did not die during their readmission.

### In-Hospital Mortality

Compared to same-hospital readmissions, older adults with AD admitted to a different hospital with a shared HIE had 39% lower odds of dying during their readmission (odds ratio [OR] 0.61, 95% CI 0.39-0.95; Table 2), accounting for readmission hospital fixed effects (not shown in table), demographics, clinical characteristics, and hospital characteristics. Beneficiaries with fragmented/different HIE readmissions had no statistically significant difference in the odds of dying during the readmission compared to same-hospital readmissions (adjusted odds ratio [AOR] 1.02, 95% CI 0.82-1.28). Beneficiaries with fragmented/no HIE had 33% increased odds of in-hospital mortality compared to same-hospital readmissions when adjusting for hospital characteristics only (AOR 1.33, 95% CI 1.01-1.75); however, the difference did not remain statistically significant when patient demographics and clinical characteristics were included (AOR 1.25, 95% CI 0.93-1.68).

**Table 2:** Unadjusted and logistic regressions for in-hospital mortality across categories of information sharing among Medicare beneficiaries with Alzheimer disease in 2018. All analyses are compared to same-hospital readmission pairs. Each model includes readmission hospital fixed effects; robust standard errors are clustered at the level of the hospital. Model 1: demographics (age, sex, race) and clinical characteristics (frailty score, chronic condition count, reason for readmission, intensive care unit stay during readmission); model 2: hospital characteristics (urban/rural, size, ownership, type, teaching status, each for readmission hospital); model 3: full model.

	Unadjusted OR^a,b^ (95% CI)	Model 1 (n=18,072), adjusted OR (95% CI)	Model 2 (n=18,157), adjusted OR (95% CI)	Model 3 (n=18,036), adjusted OR (95% CI)
Fragmented/same HIE^c^	0.72 (0.47-1.12)	0.61 (0.39-0.95)	0.72 (0.47-1.12)	0.61 (0.39-0.95)
Fragmented/different HIEs	1.07 (0.87-1.33)	1.01 (0.80-1.27)	1.09 (0.88-1.34)	1.02 (0.82-1.28)
Fragmented/no HIE participation	1.31 (0.99-1.73)	1.24 (0.92-1.66)	1.33 (1.01-1.75)	1.25 (0.93-1.68)

^a^OR: odds ratio.

^b^n=18,196.

^c^HIE: health information exchange.

### Thirty-Day Postdischarge Mortality

In unadjusted and adjusted regression models examining the odds of dying in the 30 days following hospital readmission compared to same-hospital readmission, no category of information sharing in fragmented readmissions was associated with postdischarge mortality (Table 3; hospital fixed effects not shown). However, admission-readmission pairs to different hospitals that participated in different HIEs trended toward significance when the model included hospital characteristics only (AOR 1.13, 95% CI 0.99-1.29; *P*=.06), but these results were not statistically significant.

**Table 3:** Unadjusted and logistic regressions for postdischarge mortality across categories of information sharing among Medicare beneficiaries with Alzheimer disease in 2018. All analyses are compared to same-hospital readmission pairs. Each model includes readmission hospital fixed effects; robust standard errors are clustered at the level of the hospital. Model 1: demographics (age, sex, race) and clinical characteristics (frailty score, chronic condition count, reason for readmission, intensive care unit stay during readmission); model 2: hospital characteristics (urban/rural, size, ownership, type, teaching status, each for readmission hospital); model 3: full model.

	Unadjusted OR^a,b^ (95% CI)	Model 1 (n=25,668), adjusted OR (95% CI)	Model 2 (n=25,772), adjusted OR (95% CI)	Model 3 (n=25,569), adjusted OR (95% CI)
Fragmented/same HIE^c^	1.00 (0.78-1.28)	0.95 (0.73-1.22)	1.00 (0.78-1.28)	0.95 (0.74-1.23)
Fragmented/different HIEs	1.13 (0.99-1.29)	1.11 (0.97-1.27)	1.13 (0.99-1.29)	1.11 (0.97-1.27)
Fragmented/no HIE participation	1.12 (0.94-1.33)	1.09 (0.91-1.31)	1.12 (0.94-1.33)	1.09 (0.91-1.31)

^a^OR: odds ratio.

^b^n=28,874.

^c^HIE: health information exchange.

### Sensitivity Analyses

When rural hospitals were removed to examine the effect of differences between metropolitan or micropolitan and rural market forces, the results of the analyses for both in-hospital and postdischarge mortality were similar to the primary analysis (Multimedia Appendix 4, Tables S1 and S2). When we removed beneficiaries with a probability of 90-day mortality <0.25, the association between fragmented/same HIE and lower odds of in-hospital mortality did not reach statistical significance, likely due to being underpowered (AOR 0.59, 95% CI 0.33-1.07; Multimedia Appendix 4, Table S5); all other associations were similar to the primary analysis (Multimedia Appendix 4, Tables S3-S10). Notably, when we created groups matched on the probably of mortality across information-sharing categories, the primary finding of lower in-hospital mortality remained (AOR 0.61, 95% CI 0.39-0.95; Multimedia Appendix 4, Table S7).

## Discussion

### Principal Findings

In this study, we sought to measure the association between HIE availability at the hospital level and in-hospital and postdischarge mortality following readmissions to a different hospital among older adults with AD. Compared to readmissions to the same hospital, a shared HIE during fragmented readmissions was associated with 39% lower in-hospital mortality. This benefit did not extend past the hospitalization, as there were no differences observed across the categories of HIE sharing for postdischarge mortality.

### Comparison to Prior Work

There have been a limited number of previous studies examining the relationship between HIE presence and inpatient mortality [32]: one focused on patients admitted with acute myocardial infarction found no benefit of HIEs [15], while another focusing on HIE use in interhospital transfers found a 25% lower odds of inpatient mortality [33]. Our study advances the literature in two key ways: first, by focusing on a patient population that may stand to gain outsized benefit from information exchange, and second, by using a more focused definition of information exchange—namely, whether or not the admitting and readmitting hospital participated in the same HIE—to reduce misclassification bias.

### Strengths and Limitations

This study has several limitations. First, one important unmeasured potential confounder of the relationship between electronic information exchange and mortality among older adults with AD is the presence of a caregiver. Because caregiver status and their presence during a hospital admission is not available in Medicare claims data, we were not able to assess whether caregivers were more or less likely to go to the hospital with their loved ones during fragmented or nonfragmented readmissions; and if they were present, it is unclear whether they might have served as a type of “human information exchange” for the care team. Qualitative data regarding patient and caregiver perceptions of fragmented versus nonfragmented readmissions, as well as patient and caregiver views on their role in transmitting information across fragmented settings of care, would help address this question.

Second, as with any study where HIE availability is used as a proxy for HIE use by providers, we do not know whether providers accessed the outside information, the quality or content of data shared via electronic information exchange, and, if providers accessed the data, when and how they incorporated the data into their clinical decision-making; this limitation is shared with many other studies of HIEs [13,15,33,34]. Previous work has suggested that providers do not often access HIEs [35,36], mainly because they do not perceive that HIEs contain clinically useful information [37]. Even if HIEs were accessed, we do not know how providers used the information obtained from the HIEs. Perhaps they accessed advance directives and adjusted their care plans to reflect patients’ wishes, or perhaps they reviewed old results and images that could lead to anchoring bias and potential missed diagnoses for the patient. Because the Office of the National Coordinator for Health Information Technology has made HIE availability a priority [38], HIE prevalence and use in clinical decision-making will likely grow over the coming years, and work should continue to understand how and when information contained within HIEs impacts patient outcomes. Further investigation into actual provider use of HIE at the point of care will allow researchers to go beyond HIE presence as a proxy for HIE use and will facilitate measurement of the association between how HIEs are used and patient outcomes.

### Conclusions

Overall, this study contributes to our understanding of the impact and limitations of HIEs as tools to mitigate the information discontinuity present in fragmented readmissions. It also furthers our understanding of the impact of care fragmentation on older adults with AD and how we can harness existing systems within the health IT infrastructure to lessen the effects of interhospital fragmentation of care in this population.

## Appendix

Multimedia Appendix 1 ICD-10 and DRG Codes Used for Index Admissions.

Multimedia Appendix 2 Illustration of Admission-Readmission Pairs.

Multimedia Appendix 3 Characteristics of Hospitals by Availability of Health Information Exchange, 2018 American Hospital Association Annual Survey and 2017-2018 American Hospital Association Information Technology Supplement.

Multimedia Appendix 4 Results of Sensitivity Analyses.

## Abbreviations

AD: Alzheimer disease
AHA: American Hospital Association
AMI: myocardial infarction
CHF: congestive heart failure
CMS: Centers for Medicare and Medicaid Services
COPD: chronic obstructive pulmonary disease
HIE: health information exchange
HRRP: Hospital Readmissions Reduction Program
ICU: intensive care unit
IT: information technology
MedPar: Medicare Provider Analysis and Review
UTI: urinary tract infection

## Data Availability

The data sets analyzed during the current study are not publicly available due to the data use agreement required by the Research Data Access Center/Centers for Medicare and Medicaid Services for use of identifiable Medicare data. Data are available from the corresponding author upon reasonable request and with permission of the Research Data Access Center.

## References

[1] Aldridge MD, Bradley EH (2017). Epidemiology and patterns of care at the end of life: Rising complexity, shifts in care patterns and sites of death. Health Aff (Millwood).

[2] Lin P, Zhu Y, Olchanski N, Cohen JT, Neumann PJ, Faul JD, Fillit HM, Freund KM (2022). Racial and ethnic differences in hospice use and hospitalizations at end-of-life among Medicare beneficiaries with dementia. JAMA Netw Open.

[3] Institute of Medicine (2015). Dying in America: Improving Quality and Honoring Individual Preferences Near the End of Life.

[4] Han JH, Bryce SN, Ely EW, Kripalani S, Morandi A, Shintani A, Jackson JC, Storrow AB, Dittus RS, Schnelle J (2011). The effect of cognitive impairment on the accuracy of the presenting complaint and discharge instruction comprehension in older emergency department patients. Ann Emerg Med.

[5] Callisaya M, Purvis T, Lawler K, Brodtmann A, Cadilhac D, Kilkenny M (2021). Dementia is associated with poorer quality of care and outcomes after stroke: An observational study. J Gerontol A Biol Sci Med Sci.

[6] Hustey FM, Meldon SW, Smith MD, Lex CK (2003). The effect of mental status screening on the care of elderly emergency department patients. Ann Emerg Med.

[7] Staples JA, Thiruchelvam D, Redelmeier DA (2014). Site of hospital readmission and mortality: a population-based retrospective cohort study. CMAJ Open.

[8] Tsai TC, Orav EJ, Jha AK (2015). Care fragmentation in the postdischarge period: surgical readmissions, distance of travel, and postoperative mortality. JAMA Surg.

[9] Turbow Sara, Sudharsanan Nikkil, Rask Kimberly J, Ali Mohammed K (2021). Association between interhospital care fragmentation, readmission diagnosis, and outcomes. Am J Manag Care.

[10] Brooke BS, Goodney PP, Kraiss LW, Gottlieb DJ, Samore MH, Finlayson SRG (2015). Readmission destination and risk of mortality after major surgery: an observational cohort study. Lancet.

[11] Holmgren AJ, Adler-Milstein Julia (2017). Health information exchange in US hospitals: The current landscape and a path to improved information sharing. J Hosp Med.

[12] Kuperman Gilad J (2011). Health-information exchange: why are we doing it, and what are we doing?. J Am Med Inform Assoc.

[13] Vest J, Unruh M, Freedman S, Simon K (2019). Health systems' use of enterprise health information exchange vs single electronic health record vendor environments and unplanned readmissions. J Am Med Inform Assoc.

[14] Ben-Assuli O, Shabtai I, Leshno M (2015). Using electronic health record systems to optimize admission decisions: the Creatinine case study. Health Informatics J.

[15] Chen M, Guo S, Tan X (2019). Does health information exchange improve patient outcomes? Empirical evidence from Florida hospitals. Health Aff (Millwood).

[16] Jung H, Vest JR, Unruh MA, Kern LM, Kaushal R, HITEC Investigators (2015). Use of health information exchange and repeat imaging costs. J Am Coll Radiol.

[17] Bailey JE, Pope RA, Elliott EC, Wan JY, Waters TM, Frisse ME (2013). Health information exchange reduces repeated diagnostic imaging for back pain. Ann Emerg Med.

[18] Bailey JE, Wan JY, Mabry LM, Landy SH, Pope RA, Waters TM, Frisse ME (2013). Does health information exchange reduce unnecessary neuroimaging and improve quality of headache care in the emergency department?. J Gen Intern Med.

[19] Lammers Eric J, Adler-Milstein Julia, Kocher Keith E (2014). Does health information exchange reduce redundant imaging? Evidence from emergency departments. Med Care.

[20] Hebel E, Middleton Blackford, Shubina Maria, Turchin Alexander (2012). Bridging the chasm: effect of health information exchange on volume of laboratory testing. Arch Intern Med.

[21] (2018). American Hospital Association Annual Survey. American Hospital Association.

[22] (2018). American Hospital Association Information Technology Survey. American Hospital Association.

[23] Everson J, Lee SD, Friedman CP (2014). Reliability and validity of the American Hospital Association's national longitudinal survey of health information technology adoption. J Am Med Inform Assoc.

[24] Hospital Readmissions Reduction Program: Fiscal Year (FY) 2019 Fact Sheet. Centers for Medicare and Medicaid Services.

[25] McIlvennan CK, Eapen ZJ, Allen LA (2015). Hospital readmissions reduction program. Circulation.

[26] Rudolph James L, Zanin Nicole M, Jones Richard N, Marcantonio Edward R, Fong Tamara G, Yang Frances M, Yap Liang, Inouye Sharon K (2010). Hospitalization in community-dwelling persons with Alzheimer's disease: frequency and causes. J Am Geriatr Soc.

[27] Anderson TS, Marcantonio ER, McCarthy EP, Herzig SJ (2020). National trends in potentially preventable hospitalizations of older adults with dementia. J Am Geriatr Soc.

[28] Centers for Medicare and Medicaid Services (2018). Master Beneficiary Summary.

[29] Gautam N, Bessette L, Pawar A, Levin R, Kim D (2021). Updating International Classification of Diseases 9th Revision to 10th Revision of a claims-based frailty index. J Gerontol A Biol Sci Med Sci.

[30] Kim D, Schneeweiss S, Glynn R, Lipsitz L, Rockwood K, Avorn J (2018). Measuring frailty in Medicare data: Development and validation of a claims-based frailty index. J Gerontol A Biol Sci Med Sci.

[31] Rural-Urban Continuum Codes. United States Department of Agriculture Economic Research Service.

[32] Dupont S, Nemeth J, Turbow S (2022). Effects of health information exchanges in the adult inpatient setting: A systematic review. J Gen Intern Med.

[33] Usher M, Sahni N, Herrigel D, Simon G, Melton GB, Joseph A, Olson A (2018). Diagnostic discordance, health information exchange, and inter-hospital transfer outcomes: A population study. J Gen Intern Med.

[34] Daniel OU (2018). Effects of health information technology and health information exchanges on readmissions and length of stay. Health Policy Technol.

[35] Rudin RS, Motala A, Goldzweig CL, Shekelle PG (2014). Usage and effect of health information exchange: a systematic review. Ann Intern Med.

[36] Eden KB, Totten AM, Kassakian SZ, Gorman PN, McDonagh MS, Devine B, Pappas M, Daeges M, Woods S, Hersh WR (2016). Barriers and facilitators to exchanging health information: a systematic review. Int J Med Inform.

[37] Flaks-Manov Natalie, Shadmi E, Hoshen M, Balicer RD (2016). Health information exchange systems and length of stay in readmissions to a different hospital. J Hosp Med.

[38] Health IT and Health Information Exchange Basics. Official Website of The Office of the National Coordinator for Health Information Technology.

