# Supplementary material for: The Impact of Health Information Exchange on In-Hospital and Postdischarge Mortality in Older Adults with Alzheimer Disease Readmitted to a Different Hospital Within 30 Days of Discharge: Cohort Study of Medicare Beneficiaries
Source: JMIR Aging. 2023 Mar 10;6:e41936. doi: 10.2196/41936 (PMC10039413; doi:10.2196/41936)
Supplement: Multimedia Appendix 1 [file aging_v6i1e41936_app1.docx]

**Appendix 1: ICD-10 and DRG Codes Used for Index Admissions**

**ICD-10 Codes**

| I2101 |  |
| --- | --- |
| I2102 |  |
| I2109 |  |
| I2111 |  |
| I2119 |  |
| I2121 |  |
| I2129 |  |
| I213 |  |
| I214 |  |
| I220 |  |
| I221 |  |
| I222 |  |
| I228 |  |
| I229 |  |
| I0981 |  |
| I110 |  |
| I130 |  |
| I132 |  |
| I501 |  |
| I5020 |  |
| I5021 |  |
| I5022 |  |
| I5023 |  |
| I5030 |  |
| I5031 |  |
| I5032 |  |
| I5033 |  |
| I5040 |  |
| I5041 |  |
| I5042 |  |
| I5043 |  |
| I509 |  |
| R570 |  |
| R579 |  |
| J411 |  |
| J418 |  |
| J42 |  |
| J430 |  |
| J431 |  |
| J432 |  |
| J438 |  |
| J439 |  |
| J440 |  |
| J441 |  |
| J449 |  |
| J470 |  |
| J471 |  |
| J479 |  |
| J684 |  |
| J688 |  |
| J689 |  |
| Q334 |  |
| B330 |  |
| J09X1 |  |
| J09X2 |  |
| J1000 |  |
| J1001 |  |
| J1008 |  |
| J101 |  |
| J1100 |  |
| J1108 |  |
| J120 |  |
| J121 |  |
| J122 |  |
| J123 |  |
| J1281 |  |
| J1289 |  |
| J129 |  |
| J13 |  |
| J14 |  |
| J153 |  |
| J154 |  |
| J157 |  |
| J159 |  |
| J160 |  |
| J168 |  |
| J180 |  |
| J181 |  |
| J188 |  |
| J189 |  |
| J920 |  |
| J929 |  |
| J941 |  |
| J949 |  |
| R091 |  |
| E860 |  |
| E861 |  |
| E869 |  |
| E870 |  |
| I951 |  |
| I952 |  |
| I953 |  |
| I9581 |  |
| R55 |  |
| A1810 |  |
| A1811 |  |
| A1812 |  |
| A1813 |  |
| A3685 |  |
| A5275 |  |
| A5401 |  |
| A5611 |  |
| A5619 |  |
| A985 |  |
| B650 |  |
| B901 |  |
| N10 |  |
| N110 |  |
| N118 |  |
| N119 |  |
| N12 |  |
| N135 |  |
| N136 |  |
| N151 |  |
| N2884 |  |
| N2885 |  |
| N2886 |  |
| N3000 |  |
| N3001 |  |
| N3010 |  |
| N3011 |  |
| N3020 |  |
| N3021 |  |
| N3030 |  |
| N3031 |  |
| N3080 |  |
| N3081 |  |
| N3090 |  |
| N3091 |  |
| N340 |  |
| N342 |  |
| N343 |  |
| N390 |  |
| F05 |  |
| R442 |  |
| R443 |  |

**DRGs**

| 190 |
| --- |
| 191 |
| 192 |
| 193 |
| 194 |
| 195 |
| 280 |
| 281 |
| 282 |
| 283 |
| 284 |
| 285 |
| 291 |
| 292 |
| 293 |
| 312 |
| 640 |
| 641 |
| 689 |
| 690 |
| 880 |
| 881 |
