# Supplementary figures and images for: The Impact of Health Information Exchange on In-Hospital and Postdischarge Mortality in Older Adults with Alzheimer Disease Readmitted to a Different Hospital Within 30 Days of Discharge: Cohort Study of Medicare Beneficiaries
Source: JMIR Aging. 2023 Mar 10;6:e41936. doi: 10.2196/41936 (PMC10039413; doi:10.2196/41936)

**Appendix 2: Illustration of Admission-Readmission Pairs**


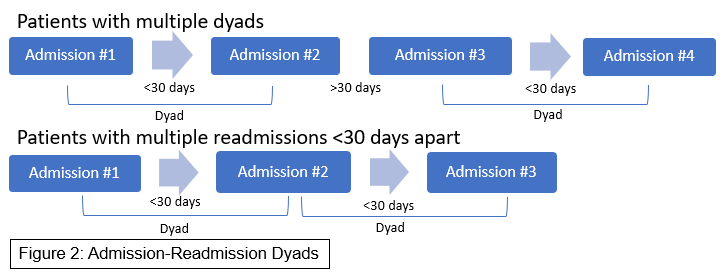

Supplement: Multimedia Appendix 2 [file aging_v6i1e41936_app2.docx]
