# Supplementary material for: The Impact of Health Information Exchange on In-Hospital and Postdischarge Mortality in Older Adults with Alzheimer Disease Readmitted to a Different Hospital Within 30 Days of Discharge: Cohort Study of Medicare Beneficiaries
Source: JMIR Aging. 2023 Mar 10;6:e41936. doi: 10.2196/41936 (PMC10039413; doi:10.2196/41936)
Supplement: Multimedia Appendix 3 [file aging_v6i1e41936_app3.docx]

**Appendix 3: Characteristics of Hospitals by Availability of Health Information Exchange, 2018 American Hospital Association Annual Survey and 2017-2018 American Hospital Association Information Technology Supplement**

|  |  | Total (n=6,292) | No Health Information Exchange  (n=1,312) | Health Information Exchange Present (n=3,142) | Health Information Exchange Information Missing (n=1,856) | p-value |
| --- | --- | --- | --- | --- | --- | --- |
| Region | **North** | 12.9% | 14.8% | 56.7% | 28.5% | <0.0001 |
|  | **South** | 39.7% | 23.4% | 43.9% | 32.7% |  |
|  | **Midwest** | 27.2% | 20.5% | 58.1% | 21.5% |  |
|  | **West** | 19.1% | 17.0% | 47.3% | 35.7% |  |
| Bed Size | **6-99 beds** | 55.3% | 23.2% | 42.7% | 34.1% | <0.0001 |
|  | **100-199 beds** | 20.1% | 20.0% | 49.7% | 30.3% |  |
|  | **200-299 beds** | 10.5% | 17.9% | 56.8% | 25.3% |  |
|  | **300-399 beds** | 5.8% | 14.5% | 66.8% | 18.7% |  |
|  | **400-499 beds** | 3.1% | 13.9% | 71.1% | 15.0% |  |
|  | **500 or more beds** | 5.2% | 10.2% | 77.1% | 12.7% |  |
| Hospital Ownership | **Government** | 22.1% | 28.0% | 38.2% | 33.9% | <0.0001 |
|  | **Church** | 8.4% | 8.2% | 73.3% | 18.5% |  |
|  | **Other not-for-profit** | 42.4% | 16.5% | 64.1% | 19.4% |  |
|  | **For-profit** | 27.1% | 25.0% | 31.0% | 44.0% |  |
| Hospital Control | **General Medical/Surgical** | 76.5% | 17.5% | 58.8% | 23.7% | <0.0001 |
|  | **Other** | 25.5% | 30.5% | 23.6% | 45.9% |  |
| Urban/Rural Status | **Metro** | 68.3% | 19.4% | 49.1% | 31.5% | <0.0001 |
|  | **Micro** | 14.0% | 20.4% | 55.6% | 24.0% |  |
|  | **Rural** | 17.7% | 25.1% | 47.0% | 27.8% |  |
| Teaching Status | **Teaching** | 38.6% | 15.8% | 59.5% | 24.7% | <0.0001 |
|  | **Non-Teaching** | 61.4% | 24.0% | 43.5% | 32.5% |  |
