# Supplementary material for: The Impact of Health Information Exchange on In-Hospital and Postdischarge Mortality in Older Adults with Alzheimer Disease Readmitted to a Different Hospital Within 30 Days of Discharge: Cohort Study of Medicare Beneficiaries
Source: JMIR Aging. 2023 Mar 10;6:e41936. doi: 10.2196/41936 (PMC10039413; doi:10.2196/41936)
Supplement: Multimedia Appendix 4 [file aging_v6i1e41936_app4.docx]

**Appendix 4: Results of Sensitivity Analyses**

**Remove admission-readmission pairs readmitted to rural hospitals**

**Appendix Table 1: Unadjusted and Logistic Regressions for In-Hospital Mortality across Categories of Information Sharing among Medicare Beneficiaries with Alzheimer’s Disease, 2018**

|  | Unadjusted^a^  n=17,988 | Model 1  n=17,864 | Model 2  N=17,949 | Model 3  N=17,828 |
| --- | --- | --- | --- | --- |
| Same HIE | 0.68 (0.44, 1.06) | 0.57 (0.36, 0.91) | 0.68 (0.44, 1.06) | 0.57 (0.36, 0.91) |
| Different HIEs | 1.08 (0.87, 1.33) | 1.00 (0.80, 1.26) | 1.08 (0.88, 1.34) | - 1. (0.81, 1.28) |
| No HIE | 1.34 (1.01, 1.76) | 1.27 (0.94, 1.70) | 1.36 (1.03, 1.78) | 1.28 (0.96, 1.72) |

1. *All analyses are compared to same hospital/nonfragmented readmission pairs. Each model includes readmission hospital fixed effects; robust standard errors are clustered at the level of the hospital.*

*Model 1: demographics (age, sex, race), clinical (frailty score, chronic condition count, reason for readmission, ICU stay during readmission)*

*Model 2: hospital (urban/rural, size, ownership, type, teaching, each for readmission hospital)*

*Model 3: Full Model*

**Appendix Table 2: Unadjusted and Logistic Regressions for Post-Discharge Mortality across Categories of Information Sharing among Medicare Beneficiaries with Alzheimer’s Disease, 2018**

|  | Unadjusted^a^  N=25,427 | Model 1  N=25,225 | Model 2  N=25,325 | Model 3  N=25,126 |
| --- | --- | --- | --- | --- |
| Same HIE | 0.97 (0.76, 1.25) | 0.92 (0.71, 1.19) | 0.97 (0.76, 1.25) | 0.92 (0.72, 1.19) |
| Different HIEs | 1.14 (1.00, 1.30) | 1.11 (0.97, 1.27) | 1.14 (1.00, 1.30) | - 1. (0.98, 1.27) |
| No HIE | 1.13 (0.95, 1.34) | 1.10 (0.92, 1.32) | 1.13 (0.95, 1.34) | 1.10 (0.92, 1.32) |

1. *All analyses are compared to same hospital/nonfragmented readmission pairs. Each model includes readmission hospital fixed effects; robust standard errors are clustered at the level of the hospital.*

*Model 1: demographics (age, sex, race), clinical (frailty score, chronic condition count, reason for readmission, ICU stay during readmission)*

*Model 2: hospital (urban/rural, size, ownership, type, teaching, each for readmission hospital)*

*Model 3: Full Model*

**Admission-readmission pairs with index ICU stay removed**

**Appendix Table 3: Unadjusted and Logistic Regressions for In-Hospital Mortality across Categories of Information Sharing among Medicare Beneficiaries with Alzheimer’s Disease, 2018**

|  | Unadjusted^a^  n=11,023 | Model 1  n=10,915 | Model 2  N=10,996 | Model 3  N=10,891 |
| --- | --- | --- | --- | --- |
| Same HIE | 0.73 (0.43, 1.26) | 0.56 (0.32, 1.00) (p=0.05) | 0.73 (0.43, 1.26) | 0.56 (0.32, 1.00) (p=0.05) |
| Different HIEs | 1.14 (0.88, 1.50) | 1.00 (0.72, 1.35) | 1.15 (0.88, 1.51) | 1.01 (0.74, 1.38) |
| No HIE | 1.05 (0.71, 1.56) | 0.93 (0.61, 1.44) | 1.07 (0.72, 1.59) | 0.95 (0.61, 1.46) |

1. *All analyses are compared to pairs in which one or both hospitals do not participate in a health information exchange (HIE). Each model includes readmission hospital fixed effects; robust standard errors are clustered at the level of the hospital.*

*Model 1: demographics (age, sex, race), clinical (frailty score, chronic condition count, reason for readmission, ICU stay during readmission)*

*Model 2: hospital (urban/rural, size, ownership, type, teaching, each for readmission hospital)*

*Model 3: Full Model*

**Appendix Table 4: Unadjusted and Logistic Regressions for Post-Discharge Mortality across Categories of Information Sharing among Medicare Beneficiaries with Alzheimer’s Disease, 2018**

|  | Unadjusted^a^  N=17,146 | Model 1  N=16,991 | Model 2  N=17,075 | Model 3  N=16,923 |
| --- | --- | --- | --- | --- |
| Same HIE | 1.08 (0.79, 1.48) | 1.02 (0.73, 1.42) | 1.08 (0.79, 1.48) | 1.02 (0.73, 1.42) |
| Different HIEs | 1.18 (1.00, 1.38) (p=0.05) | 1.10 (0.93, 1.31) | 1.17 (0.99, 1.38) (p=0.06) | 1.11 (0.93, 1.31) |
| No HIE | 1.04 (0.82, 1.33) | 0.95 (0.74, 1.23) | 1.05 (0.83, 1.34) | 0.96 (0.75, 1.24) |

1. *All analyses are compared to same hospital/nonfragmented readmission pairs. Each model includes readmission hospital fixed effects; robust standard errors are clustered at the level of the hospital.*

*Model 1: demographics (age, sex, race), clinical (frailty score, chronic condition count, reason for readmission, ICU stay during readmission)*

*Model 2: hospital (urban/rural, size, ownership, type, teaching, each for readmission hospital)*

*Model 3: Full Model*

**Remove beneficiaries with probability of 90-day mortality <0.25**

**Appendix Table 5: Unadjusted and Logistic Regressions for In-Hospital Mortality across Categories of Information Sharing among Medicare Beneficiaries with Alzheimer’s Disease, 2018**

|  | Unadjusted^a^  n=9,012 | Model 1  n=9,012 | Model 2  N=9,012 | Model 3  N=9,012 |
| --- | --- | --- | --- | --- |
| Same HIE | 0.68 (0.38, 1.20) | 0.59 (0.33, 1.07) | 0.68 (0.38, 1.20) | 0.59 (0.33, 1.07) |
| Different HIEs | 1.23 (0.95, 1.61) | 1.16 (0.89, 1.53) | 1.23 (0.95, 1.61) | - 1. (0.89, 1.53) |
| No HIE | 1.18 (0.83, 1.67) | 1.08 (0.75, 1.55) | 1.18 (0.83, 1.67) | 1.08 (0.75, 1.55) |

1. *All analyses are compared to same hospital/nonfragmented readmission pairs. Each model includes readmission hospital fixed effects; robust standard errors are clustered at the level of the hospital.*

*Model 1: demographics (age, sex, race), clinical (frailty score, chronic condition count, reason for readmission, ICU stay during readmission)*

*Model 2: hospital (urban/rural, size, ownership, type, teaching, each for readmission hospital)*

*Model 3: Full Model*

**Appendix Table 6: Unadjusted and Logistic Regressions for Post-Discharge Mortality across Categories of Information Sharing among Medicare Beneficiaries with Alzheimer’s Disease, 2018**

|  | Unadjusted^a^  N=13,332 | Model 1  N=13,332 | Model 2  N=13,332 | Model 3  N=13,332 |
| --- | --- | --- | --- | --- |
| Same HIE | 1.04 (0.76, 1.44) | 0.96 (0.69, 1.34) | 1.04 (0.76, 1.44) | 0.96 (0.69, 1.34) |
| Different HIEs | 1.23 (1.04, 1.45) | 1.19 (1.00, 1.41) (p=0.05) | 1.23 (1.04, 1.45) | 1.19 (1.00, 1.41) (p=0.05) |
| No HIE | 1.06 (0.85, 1.33) | 1.02 (0.82, 1.28) | 1.06 (0.85, 1.33) | 1.02 (0.82, 1.28) |

1. *All analyses are compared to same hospital/nonfragmented readmission pairs. Each model includes readmission hospital fixed effects; robust standard errors are clustered at the level of the hospital.*

*Model 1: demographics (age, sex, race), clinical (frailty score, chronic condition count, reason for readmission, ICU stay during readmission)*

*Model 2: hospital (urban/rural, size, ownership, type, teaching, each for readmission hospital)*

*Model 3: Full Model*

**Propensity Score Matched on odds of 30-day post dc mortality; optimal matching without replacement in 1:2 treated:control ratio, caliper=0.25**

**Appendix Table 7: Unadjusted and Logistic Regressions for In-Hospital Mortality across Categories of Information Sharing among Medicare Beneficiaries with Alzheimer’s Disease, 2018**

|  | Unadjusted^a^  n=18,196 | Model 1  n=18,072 | Model 2  N=18,157 | Model 3  N=18,036 |
| --- | --- | --- | --- | --- |
| Same HIE | 0.72 (0.47, 1.12) | 0.61 (0.39, 0.95) | 0.72 (0.47, 1.12) | 0.61 (0.39, 0.95) |
| Different HIEs | 1.07 (0.87, 1.33) | 1.01 (0.80, 1.27) | 1.08 (0.88, 1.34) | 1.02 (0.82, 1.28) |
| No HIE | 1.31 (0.99, 1.73) (p=0.06) | 1.24 (0.92, 1.66) | 1.33 (1.01, 1.75) | 1.25 (0.93, 1.68) |

1. *All analyses are compared to same hospital/nonfragmented readmission pairs. Each model includes readmission hospital fixed effects; robust standard errors are clustered at the level of the hospital.*

*Model 1: demographics (age, sex, race), clinical (frailty score, chronic condition count, reason for readmission, ICU stay during readmission)*

*Model 2: hospital (urban/rural, size, ownership, type, teaching, each for readmission hospital)*

*Model 3: Full Model*

**Appendix Table 8: Unadjusted and Logistic Regressions for Post-Discharge Mortality across Categories of Information Sharing among Medicare Beneficiaries with Alzheimer’s Disease, 2018**

|  | Unadjusted^a^  N=25,874 | Model 1  N=25,668 | Model 2  N=25,772 | Model 3  N=25,569 |
| --- | --- | --- | --- | --- |
| Same HIE | 1.00 (0.78, 1.28) | 0.95 (0.73, 1.22) | 1.00 (0.78, 1.28) | 0.95 (0.74, 1.23) |
| Different HIEs | 1.13 (0.99 1.29) (p=0.06) | 1.11 (0.97, 1.27) | 1.13 (0.99, 1.29) | 1.11 (0.97, 1.27) |
| No HIE | 1.12 (0.94, 1.33) | 1.09 (0.91, 1.31) | 1.12 (0.94, 1.33) | 1.09 (0.91, 1.31) |

1. *All analyses are compared to same hospital/nonfragmented readmission pairs. Each model includes readmission hospital fixed effects; robust standard errors are clustered at the level of the hospital.*

*Model 1: demographics (age, sex, race), clinical (frailty score, chronic condition count, reason for readmission, ICU stay during readmission)*

*Model 2: hospital (urban/rural, size, ownership, type, teaching, each for readmission hospital)*

*Model 3: Full Model*

**Removed second and beyond pairs for beneficiaries with only same-hospital readmissions**

**Appendix Table 9: Unadjusted and Logistic Regressions for In-Hospital Mortality across Categories of Information Sharing among Medicare Beneficiaries with Alzheimer’s Disease, 2018**

|  | Unadjusted^a^  n=18,111 | Model 1  n=18,059 | Model 2  N=18,075 | Model 3  N=18,023 |
| --- | --- | --- | --- | --- |
| Same HIE | 0.72 (0.47, 1.11) | 0.61 (0.39, 0.95) | 0.72 (0.47, 1.11) | 0.61 (0.39, 0.95) |
| Different HIEs | 1.06 (0.86, 1.32) | 1.01 (0.80, 1.27) | 1.07 (0.87, 1.33) | - 1. (0.82, 1.28) |
| No HIE | 1.30 (0.99, 1.71) (p=0.06) | 1.24 (0.92, 1.66) | 1.32 (1.00, 1.74) | 1.25 (0.93, 1.68) |

1. *All analyses are compared to same hospital/nonfragmented readmission pairs. Each model includes readmission hospital fixed effects; robust standard errors are clustered at the level of the hospital.*

*Model 1: demographics (age, sex, race), clinical (frailty score, chronic condition count, reason for readmission, ICU stay during readmission)*

*Model 2: hospital (urban/rural, size, ownership, type, teaching, each for readmission hospital)*

*Model 3: Full Model*

**Appendix Table 10: Unadjusted and Logistic Regressions for Post-Discharge Mortality across Categories of Information Sharing among Medicare Beneficiaries with Alzheimer’s Disease, 2018**

|  | Unadjusted^a^  N=25,744 | Model 1  N=25,648 | Model 2  N=25,645 | Model 3  N=25,549 |
| --- | --- | --- | --- | --- |
| Same HIE | 0.99 (0.77, 1.27) | 0.95 (0.73, 1.22) | 0.99 (0.78, 1.27) | 0.95 (0.74, 1.23) |
| Different HIEs | 1.12 (0.99, 1.28) (p=0.07) | 1.11 (0.97, 1.27) | 1.12 (0.99, 1.28) | - 1. (0.97, 1.27) |
| No HIE | 1.11 (0.94, 1.32) | 1.09 (0.91, 1.31) | 1.12 (0.94, 1.33) | 1.09 (0.91, 1.31) |

1. *All analyses are compared to same hospital/nonfragmented readmission pairs. Each model includes readmission hospital fixed effects; robust standard errors are clustered at the level of the hospital.*

*Model 1: demographics (age, sex, race), clinical (frailty score, chronic condition count, reason for readmission, ICU stay during readmission)*

*Model 2: hospital (urban/rural, size, ownership, type, teaching, each for readmission hospital)*

*Model 3: Full Model*
